# Infection Control in Healthcare Settings in Japan

**DOI:** 10.2188/jea.JE20110085

**Published:** 2012-03-05

**Authors:** Keita Morikane

**Affiliations:** Division of Clinical Laboratory and Infection Control, Yamagata University Hospital, Yamagata, Japan

**Keywords:** healthcare-associated infection, infection control, healthcare insurance system

## Abstract

In Japan, the practice of infection control in healthcare settings has a short history of less than 3 decades. Before that, infection control practices were far from perfect and even ignored. This review summarizes changes in infection control in Japan since the 1980s and offers some comparisons with practices in foreign countries, especially the United States. Infection control is far better now than 25 years ago, but there remain fundamental issues that limit the development of better infection control practices. These problems include insufficient funding and human resources due to the socialized healthcare insurance system in Japan and the lack of interest in infection control research.

## INTRODUCTION

In Japan, infection control in the healthcare setting was not an organized endeavor until the founding of a society for infection control (Japanese Society for Environmental Infections, JSEI) in 1986.^1^ During my medical education in the mid-1980s, there was no instruction in infection control. The idea of universal precautions, which became popular in healthcare settings in Western countries, had not yet been introduced to Japan, and procedures with some risk of exposure to blood-borne pathogens, such as phlebotomy and peripheral line insertion, were often done without gloves. This was during the time when methicillin-resistant *Staphylococcus aureus* (MRSA) became prevalent throughout Japan, especially in postoperative patients. Many had diarrhea with a MRSA-positive stool culture, which was diagnosed as MRSA enterocolitis. This disease entity is still debated, but that situation led to the development of a relevant infection control strategy in Japan, something that had never existed before. The key events since 1986 are listed in Table 1. In this article, I describe the changes in infection control in Japan during the last 25 years and discuss the present situation and challenges we currently face.

**Table 1.** Key events in infection control in Japan

1980s	Increase in the incidence of healthcare-associated MRSA infection
1986	Founding of JSEI
1993	Infection control department established at The University of Tokyo Hospital (first in Japan)
1999	Nationwide surveillance of surgical site infections by JSEI
2000	Nationwide surveillance of multidrug-resistant organisms by MHLW
2000	832 MD and PhD staff certified as Infection Control Doctors (ICD)
2001	18 nurses certified as Infection Control Nurses (ICN)
2004	Mandatory assignment of dedicated infection control personnel at Advanced Treatment Hospitals
2010	Revision of medical reimbursement system: implementation of additional reimbursement for advanced infection control management

### The infection control team

For effective infection control, it is necessary to combine personnel who spend a specified fraction of their time—usually expressed in full-time equivalents (FTEs)—on infection control. Therefore, teamwork among infection control personnel is very important. The infection control team (ICT) is a very popular concept among healthcare workers in Japan, in contrast to the situation in the United States, where members of each profession (physicians, nurses, pharmacists, microbiologists, etc) work independently for infection control and are responsible for their designated, specialized areas. Although Japanese law does not require hospitals to have ICTs, they must have an infection control committee (ICC) that includes the chairperson and executive officers of the hospital. The ICC makes the final decisions regarding infection control programs, but this is often just an endorsement.

Program establishment and practice are led by the ICT, which conducts surveillance of multidrug-resistant pathogens and device/procedure-associated infections such as bloodstream infection (BSI), urinary tract infection (UTI), and surgical site infection (SSI). Other activity includes ward audit (rounds), education of healthcare personnel, adherence monitoring of hand hygiene and other infection control practices, and investigation of possible outbreaks. The ICT develops and revises infection control policy in their hospital. The work volume is too great for a single individual; thus, the ICT is essential for infection control activities.

### Surveillance

Until the mid-1990s, interest in surveillance was very low. Only a small group of people who learned and were inspired by US infection control practices conducted surveillance at the hospital level. In 1998, the JSEI established a surveillance system in Japan, the Japanese Nosocomial Infections Surveillance (JNIS). The system was based on the US National Nosocomial Infection Surveillance (NNIS) system with some modifications and initially focused on SSIs. Eight hospitals participated initially, and more hospitals joined later. Currently, approximately 50 hospitals send their data to the system each year. Aggregated data are analyzed, and feedback is sent to the hospitals by emails that provide detailed data on the respective hospitals and the overall system. Aggregated data are presented at US meetings such as those of the Society for Healthcare Epidemiology of America (SHEA) and the Infectious Diseases Society of America (IDSA). Discussion of data collection, analysis, and other issues related to SSI surveillance takes place at an SSI surveillance meeting, which has been held twice a year since 2002.

The Japanese Ministry of Health, Labour and Welfare (MHLW) administers another surveillance system, the JApan Nosocomial Infection Surveillance (JANIS), which was developed as a public health service and mainly focuses on collecting data on nosocomial infection by multidrug-resistant organisms (MDROs) such as MRSA, although the system was based on similar components in the NNIS system. The main elements are a laboratory component and a hospital-wide MDRO component. For the intensive care unit (ICU) component, hospitals collect device-associated infection data. The denominator of the infection rate was initially device-days but was changed to patient-days of the targeted ICUs. This change was based on data showing that the infection rate was similar using device-days and patient-days as the denominator in this surveillance system.^2^ However, this modification made Japanese data incompatible with data from other countries, such as the United States, where device-days remains the denominator in this form of surveillance.

The JNIS system was renamed JHAIS (Japanese Healthcare-associated Infections Surveillance) in 2008 and began monitoring device-associated infection (ie, central line-associated BSI, catheter-associated UTI, and ventilator-associated pneumonia) as well. Approximately 20 hospitals participate in this system.

### The professional community

JSEI was established in 1986 with only 231 members. In 2011, only 25 years after its founding, it has more than 6000 members. Among the 3984 members who disclose their occupation to the Society, most are registered nurses (42%). The proportions of physicians, pharmacists, and clinical laboratory technologists are 19%, 16%, and 8%, respectively. Recent annual meetings have attracted more than 5000 attendees, which exceeds the attendance of any of the 3 major Western healthcare epidemiology organizations, ie, the Association for Professionals in Infection Control and Epidemiology (APIC), SHEA in the United States, and the Hospital Infection Society (HIS) in the United Kingdom.

JSEI activity includes an annual meeting (in February) and publication of a journal (6 issues per year). It also has several committees, including editorial, educational, and international committees. The first training course for healthcare epidemiology was held in 2009 by the educational committee.

The organization responsible for the diagnosis and treatment of infectious diseases in Japan is the Japanese Society for Infectious Diseases. The Japanese Society for Chemotherapy oversees antimicrobials, and the Japanese Society for Microbiology is responsible for clinical microbiology.

### Certification

Japan has 4 specialized certifications in infection control for different occupations.

#### Certified Nurse for Infection Control (CNIC)

The Japanese Nursing Association accredits this certification, which requires 6 months of intensive study at a designated educational institution in Japan and a passing grade on the certification examination. Eighteen nurses were certified after the first examination, in 2001, and as of July 2011 there are 1364 CNICs in Japan. In 2011, 10 institutions offer education for this certification. The curriculum in infection control is comprehensive and includes surveillance, practice, microbiology, and planning of infection control programs in healthcare facilities.

#### Infection Control Doctor (ICD)

The Committee for the ICD accredits this certification. Candidates must be a medical doctor (MD), or have a PhD in a healthcare field, for more than 5 years, be a member of a society approved by the Committee, have experience in infection control in a healthcare setting, and have proof of participation in educational meetings or scientific conferences. An examination is not necessary for certification. A certified ICD is expected to lead infection control activities in a hospital, with the support of the members from each healthcare profession (ie, physicians, nurses, pharmacists, and microbiologists) and administrative staff. In the first year, 2000, a total of 832 persons were certified; as of January 2010, 6815 have been certified.

#### Board-Certified Infection Control Pharmacy Specialist (BCICPS)

The Japanese Society of Hospital Pharmacists (JSHP) accredits BCICPS certification. Candidates for certification must: be a licensed pharmacist, be a member of the JSHP, be a certified ICD, be named as an author in 3 abstracts for designated scientific conferences in the pharmacy field (and as first author in at least 1), have 2 publications in the field of infection control (and as first author in at least 1), and pass an examination. Forty pharmacists were certified in the first year, 2005, and 219 have been certified as of April 2011.

This certification system was aimed to designate pharmacists who are routinely involved in infection control activities, provide instruction to the next generation of pharmacists in infection control, and conduct research in the field. However, because the requirements for certification are very high, a new category was created in 2008, namely, Board-Certified Pharmacist in Infection Control (BCPIC), which is less advanced than the BCICPS. Ninety-four pharmacists were certified in the first year, and 364 have been certified as of October 2010.

#### Infection Control Microbiological Technologist (ICMT)

The Japanese Society for Clinical Microbiology (JSCM) accredits ICMT certification. Candidates must be a clinical technologist and a member of the JSCM and be active in infection control practices in a healthcare setting, among other requirements. In the first year, 2006, 253 technologists were certified, and 411 have been certified as of January 2011.

### Public organizations and agencies

The Centers for Disease Control and Prevention (CDC) in the United States and the Health Protection Agency (HPA) in the United Kingdom are 2 of the most famous organizations in the world. At the national level in Japan, infection control in healthcare settings is under the jurisdiction of the Medical Service Division, Health Policy Bureau, MHLW. This division has technical and administrative officers whose main role is to manage rules and regulations, such as laws and bylaws. The National Institute of Infectious Diseases (NIID) is expected to support the MHLW both technically and scientifically and is equivalent to the US CDC and HPA. NIID is designed to function as a research laboratory for various pathogens, a reference laboratory for nationwide research on microbiology, and a laboratory for investigation of numerous drugs, blood products, and vaccines. There is no designated section in NIID for infection control in healthcare settings. It is therefore impossible to create official guidelines for healthcare-associated infections (HAIs) or lead HAI surveillance. Regarding epidemiologic investigation of HAIs, the Field Epidemiology Training Program (FETP) in the NIID investigated approximately 10 HAI outbreaks caused by pathogens such as vancomycin-resistant *Enterococcus*, multidrug-resistant *Pseudomonas aeruginosa* (MDRP), and *Clostridium difficile*. The FETP is a 2-year intensive course in field epidemiology.

### Laws and rules

The Japanese healthcare system is regulated by the Medical Service Act (*Iryou-hou* in Japanese). In the 2007 version of the Act, healthcare safety is a primary goal for every hospital and clinic. In addition, in the Ordinance for Enforcement of the Medical Service Act (*Iryou-hou shikou kisoku*), prevention of HAIs is expressly included as part of healthcare safety. Health centers must establish an HAI policy in each facility, form a committee for HAI prevention, educate employees, and take part in HAI surveillance and reporting. It also requires advanced treatment hospitals (ATHs) and teaching hospitals to establish an HAI prevention department and designate a person(s) to staff the department. This regulation is mandatory, and penalties may apply in cases of intentional violations.

### Reimbursement

The costs of HAI prevention are paid by hospitals, and, until recently, no reimbursement was given for superior HAI prevention practices. In addition, reimbursement for treatment of HAIs was equal to that of the respective infectious disease, which meant that there was no incentive to implement better HAI prevention practices.

In 1996, hospitals with good infection control practices began to receive an additional reimbursement of 50 yen (0.6 USD) per patient per day. The requirements for this additional reimbursement were minimal, so, within 2 years, 70% of Japanese hospitals had applied for it. In 2000, this reimbursement policy was discontinued and replaced with a new system of penalties for hospitals with insufficient infection control practices. This policy was also discontinued, in 2006.

In 2010, as part of healthcare safety, a reimbursement of 1000 yen (12 USD) per patient per admission was introduced. Before this policy was begun, most hospitals in Japan did not give physicians or CNICs a designated time period for infection control. If a hospital wishes to receive the reimbursement, it must pay the annual cost for the designated work hours for a physician (a salary of 0.5 full-time equivalents [FTEs], about 4 million yen) and a nurse (a salary of 0.8 FTEs, about 4 million yen), which equals approximately 8 million yen (Table 2). This is roughly equal to the amount that would be reimbursed for a hospital with 300 beds and an average length of stay of 15 days. Therefore, generally speaking, this incentive is attractive for hospitals with more than 300 beds but not for those with fewer beds. There are no data on the number of hospitals that have applied for this reimbursement.

**Table 2.** Requirements for additional reimbursement (April 2010)

Division of infection control	
Infection control team in division, consisting of the following:	
	(1) At least 1 infection control nurse with designated training experience and at least 1 infection control physician with designated infection control experience (1 with >80% FTE; the other with >50% FTE)
	(2) Infection control pharmacist and infection control microbiology technologist, both with experience in infection control
Policy regarding duties of infection control team	
Hospital infection control policy must be distributed ​ to all wards and divisions	
Educational lecture for all staff, at least twice a year	
Antimicrobial stewardship program	

### Guidelines

Due to the situation regarding the public organization that oversees infection control, there is no official guideline published by the government. The MHLW has a research fund that it is distributed to selected research groups, which create documents similar to guidelines. These are usually prepared based on guidelines published by the CDC with some modifications. Furthermore, research in this field is limited in Japan, so the country has very few data of its own. Therefore, documents published by these groups are more like expert opinions.

Scientific societies and professional organizations have important roles to play. In 1990, the JSEI published *A Guide for the Prevention of Hospital Infection*, the first publication of its kind in Japan. In 2001, the Japanese Nursing Association published *A Guidebook for Infection Control*, which was followed by the *Guideline For Hospital Infection Control* and the *ICD Textbook*, published by the Committee for National University Hospitals and the Committee for the ICD, respectively. These publications are updated regularly and are widely used in the Japanese infection control community. In addition, numerous commercial-based documents have been published.

### Education

In general, infection control personnel in hospitals are certified, experienced, and knowledgeable and are responsible for teaching infection control practices to other healthcare workers. The Health Service Act mandates education sessions in every hospital and clinic, and a supplemental document published by the MLHW specifies that the sessions should be held more than twice a year. To fulfill this requirement, large hospitals hold education sessions within their facility; however, in smaller hospitals and clinics, this might be difficult. As an alternative, they can subsidize healthcare workers to attend seminars hosted by a local government, society, university hospital, or even a private company. There are numerous seminars on infection control throughout Japan. However, because many are held in large metropolitan areas, their geographical distribution is uneven.

Education for certification in infection control is provided by each accrediting body, but there is no education in healthcare epidemiology in Japan. Many universities regard infection control as a clinical practice rather than a field of study and do not have a department of infection control in their (graduate) school of medicine. As a result, healthcare epidemiology is made light of by the medical community and is not regarded as fundamental to infection control. The JSEI held its first healthcare epidemiology training course in Japan in 2009. About 50 infection control professionals attended the session, and it will now be held annually. This might increase the number of professionals who are able to conduct high-quality studies in infection control.

### Research

Few publications by Japanese researchers in infection control have been published in English. There are several possible reasons for this, namely (1) not many universities have a department of infection control, which means that even in university hospitals, there is inadequate staffing, funding, and time allocated for research, (2) Japan’s socialized health insurance system limits staffing and funding resources for infection control, (3) there are few educational opportunities in healthcare epidemiology, and (4) although research groups receive funding from the MHLW, this funding is closely related to government policy, and the areas of interest are limited.

### The media and the general public

The media often react hysterically to clusters or outbreaks of multidrug-resistant organisms. For example, in September 2010, a university hospital in Tokyo had an outbreak of multidrug-resistant *Acinetobacter baumannii*. The media reported the case and related issues in the headlines for 1 week and then suddenly stopped covering it, presumably because they had become disinterested. The reporting during that week was full of sensationalism and lacked a scientific understanding of the situation. Indeed, the outbreak was investigated by a scientific body and the police.^3^ The Japanese authorities have a tendency to investigate events (and not only HAIs) from a punitive rather than a scientific perspective, and the general public has the same tendency. After the events described above, there were many anonymous online comments criticizing the university hospital.

### Challenges in infection control practices in healthcare settings

The most serious fundamental problem in infection control is the lack of personnel assigned to infection control in hospitals. Under the socialized medical insurance system, hospitals tend to assign healthcare personnel to areas that produce direct revenue, and infection control is not such an area.

In the United States, the standard ratio of infection control personnel is about 1 per 250 beds. The figure among hospitals participating in the National Nosocomial Infections Surveillance system is about 1 per 115 beds.^4^ However, there are limited data on personnel assigned to infection control. The MHLW conducted a survey of advanced treatment hospitals (*Tokutei Kinou Byouin*). There are 83 ATHs in Japan, and most are affiliated with faculties of medicine. According to the *Disclosure of Practice Report From ATHs*, which was published in 2009,^5^ there were 159 designated personnel in 83 ATHs that had a total of 72 178 beds. This means that, on average, there is 1 infection control specialist per 454 beds, which is far lower than the standard ratio in the United States. Among the 83 ATHs, only 10 had more than 1 specialist per 250 beds. ATHs with more than 1000 beds often had only 1 infection control specialist.

Another survey of healthcare facilities^6^ found that, as of October 2008, there were 468 hospitals with more than 500 beds. Among them, 204 (44%) had 1 or more infection control specialist with an FTE of 0.8 or greater (ie, a person almost completely concerned with infection control), and 253 (54%) had 1 or more personnel with an FTE of between 0.2 and 0.8. Eleven hospitals (2%) had no designated personnel. These data show that even in ATHs, which have the resources to assign personnel working in the faculty of medicine (ie, people not employed by the hospital) to infection control in the hospital, few personnel were actually assigned to infection control. The situation in non-AHTs is likely to be much worse in terms of human resources.

Beginning in April 2010, a new reimbursement system came into effect. This gives a hospital about 12 USD per patient if it fulfils MHLW requirements regarding infection control, which mandate an infection control nurse and infection control physician (one at 0.5 FTE; the other at 0.8 FTE), a pharmacist (0.5 FTE), and a microbiologist (0.5 FTE). This revision favors larger hospitals, which are better able to fulfill the requirement and thus receive the reimbursement. Considerable changes in infection control practices are anticipated.

### Summary

The present author’s experience visiting many US hospitals suggests that infection control practices in Japanese hospitals are as good as those in US hospitals. However, Japan is far behind in terms of research and data collection. More attention and funding are therefore required in these areas.
